# Development and Optimization of Indirect ELISAs for the Detection of Anti-Capripoxvirus Antibodies in Cattle, Sheep, and Goat Sera

**DOI:** 10.3390/microorganisms10101956

**Published:** 2022-09-30

**Authors:** Francisco J. Berguido, Esayas Gelaye, Yang Liu, Batdorj Davaasuren, Kiril Krstevski, Igor Djadjovski, Emiliya Ivanova, Gabriela Goujgoulova, Angelika Loitsch, Eeva Tuppurainen, Tesfaye Rufael Chibssa, Philippe Caufour, Milena Samojlović, Sava Lazić, Tamaš Petrović, Dejan Vidanović, Stéphane Bertagnoli, Reingard Grabherr, Adama Diallo, Giovanni Cattoli, Charles Euloge Lamien

**Affiliations:** 1 Animal Production and Health Laboratory, Animal Production and Health Section, Joint FAO/IAEA Division, Department of Nuclear Sciences and Applications, International Atomic Energy Agency, WagramerStrasse 5, P.O. Box 100, A1400 Vienna, Austria; 2 Institute of Biotechnology, University of Natural Resources and Life Sciences (BOKU), Muthgasse 18, 1190 Vienna, Austria; 3 Food and Agriculture Organization of the United Nations, Sub-Regional Office for Eastern Africa, Addis Ababa P.O. Box 5536, Ethiopia; 4 China National Clinical Research Center for Neurological Diseases, Beijing Tiantan Hospital, Capital Medical University, Beijing 100070, China; 5 Laboratory of Molecular Genetics, Institute of Veterinary Medicine, Mongolian University of Life Sciences, Ulaanbaatar 17024, Mongolia; 6 Veterinary Institute, Faculty of Veterinary Medicine, University “Ss. Cyril and Methodius”, Skopje 1000, North Macedonia; 7 National Diagnostic and Research Veterinary Institute (NDRVMI), 1231 Sofia, Bulgaria; 8 Austrian Agency for Health and Food Safety (AGES), Spargelfeldstrasse 191, 1220 Vienna, Austria; 9 Institute of International Animal Health/One Health, Friedrich-Loeffler-Institut, D-17493 Greifswald, Germany; 10 Animal Health Institute, Sebeta P.O. Box 04, Ethiopia; 11 UMR Cirad-Inrae 117 ASTRE, Department BIOS, CIRAD, 34398 Montpellier, France; 12 Scientific Veterinary Institute “Novi Sad”, 21000 Novi Sad, Serbia; 13 Veterinary Specialized Institute Kraljevo, Zicka 34, 36103 Kraljevo, Serbia; 14 Ecole Nationale Vétérinaire de Toulouse, IHAP, Université de Toulouse, INRAE, ENVT, 23 Chemin des Capelles, 31076 Toulouse, France; 15 CIRAD, UMR ASTRE, ISRA/LNERV, Dakar BP 2057, Senegal

**Keywords:** capripoxvirus, iELISA, A34, A36, LSDV, SPPV, GTPV

## Abstract

Sheeppox (SPP), goatpox (GTP), and lumpy skin disease (LSD) are economically significant pox diseases of ruminants, caused by sheeppox virus (SPPV), goatpox virus (GTPV), and lumpy skin disease virus (LSDV), respectively. SPPV and GTPV can infect both sheep and goats, while LSDV mainly affects cattle. The recent emergence of LSD in Asia and Europe and the repeated incursions of SPP in Greece, Bulgaria, and Russia highlight how these diseases can spread outside their endemic regions, stressing the urgent need to develop high-throughput serological surveillance tools. We expressed and tested two recombinant truncated proteins, the capripoxvirus homologs of the vaccinia virus C-type lectin-like protein A34 and the EEV glycoprotein A36, as antigens for an indirect ELISA (iELISA) to detect anti-capripoxvirus antibodies. Since A34 outperformed A36 by showing no cross-reactivity to anti-parapoxvirus antibodies, we optimized an A34 iELISA using two different working conditions, one for LSD in cattle and one for SPP/GTP in sheep and goats. Both displayed sound sensitivities and specificities: 98.81% and 98.72%, respectively, for the LSD iELISA, and 97.68% and 95.35%, respectively, for the SPP/GTP iELISA, and did not cross-react with anti-parapoxvirus antibodies of cattle, sheep, and goats. These assays could facilitate the implementation of capripox control programs through serosurveillance and the screening of animals for trade.

## 1. Introduction

Sheeppox virus (SPPV), goatpox virus (GTPV), and lumpy skin disease virus (LSDV) are large, double-stranded DNA viruses of the genus *Capripoxvirus* of the *Poxviridae* family [1]. Although these three viruses share high sequence similarity (96 to 97% nucleotide identity) [2,3], they display specific host preferences [4,5]. SPPV and GTPV can infect sheep and goats, causing sheeppox (SPP) and goatpox (GTP), respectively. Though most strains cause disease in sheep and goats, some strains show preferences for either one or the other species [3,6]. Additionally, GTPV infection in wildlife has recently been reported [7,8]. Likewise, LSDV causes lumpy skin disease (LSD) in cattle and buffaloes, while clinical signs have been reported in Springbok antelope, oryx, and gazelle [9].

LSD, SPP, and GTP are categorized by the World Organization for Animal Health (WOAH) as notifiable diseases [10], and are included on the list of potential bioterrorist agents by the United Kingdom and the United States Department of Agriculture [11,12].

SPP and GTP remain endemic in Asia, Africa (except for southern Africa), the Middle East, and Turkey [13,14], with sporadic outbreaks of SPP in Greece and parts of Eastern Europe [13].

LSD was endemic only in the Middle East and sub-Saharan Africa, including Egypt [15]. However, in 2015, the first outbreaks of LSD occurred in Greece, the Russian Federation, Central Asia, and most of the Balkans [13,16]. LSD was detected in the remaining part of Asia in 2019, where it is likely to become endemic [17].

Several highly efficient tests for the molecular identification and differentiation of capripoxviruses have been developed and are currently used [4,5,18,19,20,21,22,23,24,25,26]. In contrast, only a few serological assays have been described. All of them present several drawbacks. For example, the WOAH-recommended virus neutralization test (VNT) for anti-CaPV antibody detection [6,27] requires large amounts of sera, is not easy to implement, and its results can be difficult to read [9,28]. In addition, VNT also requires the use of live viruses, which limits its use to BSL-3 facilities in capripox non-endemic countries or in well-equipped virological laboratories in endemic countries. The indirect fluorescent antibody test (IFAT), also on the WOAH list of available serological tests, cross-reacts with orf (contagious pustular dermatitis) in sheep and other poxviruses [6,27]. Western blot and agar gel immunodiffusion tests can also be used in serological capripoxvirus antibody detection, but while the former is expensive to perform and not convenient in high-throughput testing [6,27], the latter cross-reacts with antibodies against parapoxviruses [6,27].

Several ELISAs have also been developed, using either purified viral preparations [29] or recombinant capripoxvirus proteins [30,31,32]. However, these ELISAs have encountered practical problems, hampering their use. For example, recombinant proteins have shown a loss of activity due to protein instability of the recombinant antigens [29] or poor protein expression levels [30]. Meanwhile, ELISAs based on inactivated viruses bear the high cost of producing large amounts of the antigen [31]. Other tests have reported problems detecting a particular category of antibodies: Tian et al. (2010) based their iELISA on two synthetic peptides of CaPV P32, but this assay performed well only on sera from immunized sheep [32]. In addition, the test by Bowden et al. (2009), targeting anti-SPPV and anti-GTPV antibodies by using CaPV core virion proteins for an indirect ELISA, performed well with experimentally infected, but not with vaccinated, sheep and goat sera samples or LSDV-infected cattle serum [33].

The present study builds on previous work performed in our laboratory [34], where several antigenic targets were analyzed in order to develop a capripoxvirus iELISA. We considered two target antigens, the capripoxvirus homologs of the vaccinia virus C-type lectin-like protein A34 and the EEV glycoprotein A36, from a panel of five surface and immunomodulatory proteins. We also compared several blocking buffers and secondary antibodies to improve binding and detection. Finally, based on those results, we established two test conditions, using the recombinant truncated capripoxvirus A34 protein as an antigen.

## 2. Materials and Methods

### 2.1. Target Sequence Selection, Cloning, and Expression of Proteins

The details of the target selection, cloning, and expression of proteins were previously described by Gelaye [34]. Briefly, the GTPV Pellor (NC_004003) homologs of the vaccinia virus C-type lectin-like protein A34 (LSDV ORF 123) and EEV glycoprotein A36 (LSDV ORF 126) were analyzed in order to identify transmembrane domains, which would render the sequences hydrophobic. Using TMHMM Server v. 2.0, the amino terminus showed the predicted presence of transmembrane and hydrophobic residues. Therefore, forward primers were designed to exclude this region.

The primers were designed containing attB site sequences both at the 5ʹ and 3ʹ sides of the fragment (Table 1) for PCR amplification using the genome sequence of GTPV Pellor (NC_004003). The targeted truncated fragments were amplified using GTPV Denizli as a template on the Bio-Rad C1000 Touch™ Thermal Cycler, and the PCR products were verified by gel electrophoresis. Cloning was performed by Gateway^®^ cloning technology (pDONR™221 and pDEST™17) (Thermo Fisher Scientific, Waltham, MA, USA), following the manufacturer’s guidelines. The final constructs in the destination vector were transformed into DH5α and BL21 competent cells (Thermo Fisher Scientific, Waltham, MA, USA). During all cloning steps, the presence of the correct inserts was verified by sequencing.

Protein expression was conducted by growing the cells in LB broth medium (Thermo Fisher Scientific, Waltham, MA, USA) containing carbenicillin (Merck KGaA, Darmstadt, Germany), and inducing the protein expression with L-arabinose (Merck KGaA, Darmstadt, Germany) for four hours. Upon mechanical lysis of the cells, the inclusion bodies were solubilized in a buffer containing 8 M urea. The recombinant peptide was purified based on the presence of a His-tag, using a HisTrap Talon column (GE Healthcare, Chicago, IL, USA), by the ÄKTA Prime Plus protein purification system (Cytiva, Marlborough, MA, USA), followed by stepwise dialysis into PBS 1% SDS with protease inhibitors (Roche, Basel, Switzerland). The proteins were analyzed using SDS-PAGE, and antibody binding, using both goat and cattle positive sera, was confirmed by Western blot. After the initial proof of concept, the proteins, based on the corresponding sequences of LSDV NI-2490 (NC_003027), were outsourced for production to GenScript, Inc. (Piscataway, NJ, USA).

### 2.2. Western Blot

The preparation of proteins for the SDS-PAGE gels and Western blots followed standard procedures. The antigen and protein preparations (20 µL) were heated at 80 °C for 10 min in 4X LDS sample buffer (Thermo Fisher Scientific, Waltham, MA, USA). The samples were loaded onto an SDS-PAGE (NUPAGE 10% (*v/v*) gel) (Thermo Fisher Scientific, Waltham, MA, USA) and transferred to a 0.2 µm PVDF membrane (Thermo Fisher Scientific, Waltham, MA, USA). The membrane was probed for 1 h at room temperature with the anti-penta His antibody (Amgen, Thousand Oaks, CA, USA) diluted 1:500 in blocking buffer, with undiluted hyper-immune KS1-positive sheep serum (kindly provided by the Austrian Agency for Health and Food Safety (AGES), Austria), or with orf-positive goat serum diluted 1:50 in blocking buffer, and then incubated overnight. The membrane that was probed with the anti-penta His antibody was washed three times with PBS containing 0.5% (*v/v*) Tween 20 and was probed for 1 h with diluted (1:5000) goat anti-mouse antibodies conjugated to horseradish peroxidase (Merck KGaA, Darmstadt, Germany). The membrane probed with KS1-positive sheep serum was washed three times with PBS containing 0.5% (*v/v*) Tween 20 and was probed for 1 h with diluted (1:1000) donkey anti-sheep antibodies conjugated to horseradish peroxidase (Merck KgaA, Darmstadt, Germany). The membrane that was probed with orf-positive goat serum was washed three times with PBS containing 0.5% (*v/v*) Tween 20 and was probed for one hour with diluted (1:30,000) rabbit anti-goat antibodies conjugated to horseradish peroxidase (Merck KgaA, Darmstadt, Germany). For detection, ECL substrate (GE Healthcare, Chicago, IL, USA) was used according to the manufacturer’s instructions.

### 2.3. Virus Neutralization Test

Briefly, in quadruplicate, heat-decomplemented (56 °C for 1 h) serum samples and positive and negative controls were diluted, starting at 1 in 16 in DMEM cell culture medium (Thermo Fisher Scientific, Waltham, MA, USA), and incubated with and without 100 TCID_50_ final concentration of wild-type LSDV Massalamia [35] for 1 h at room temperature. Then, 20,000 ESH-L cells/well were added. The plates were incubated at 37 °C for 8 days, after which they were examined for cytopathic effects (CPE). A serum sample was considered negative when CPEs were observed in at least three of four wells, and positive when CPEs were blocked in at least two out of the four wells.

### 2.4. Serum Samples

#### 2.4.1. Experimental and Field Sera

There were four types of reference population sera used for this study:

(a) Bovine LSD-positive sera (n = 78), obtained either from experimental infections (conducted at The Pirbright Institute, UK) [36] or from naturally LSDV-infected animals from Bulgaria (NDRVMI, Sofia, Bulgaria) and the Republic of North Macedonia (School of Veterinary Medicine, University “Ss Cyril and Methodius”, Skopje, Republic of North Macedonia). These samples were confirmed positive by VNT (supplementary Table S1); (b) bovine LSD-negative sera (n = 252) from countries where the disease is historically not present, namely France (provided by IDVet, Montpellier, France), Austria (provided by AGES, Austria), and the Republic of North Macedonia (sera collected before 2010) (School of Veterinary Medicine, University “Ss Cyril and Methodius”, Skopje, Republic of North Macedonia); (c) Capripox-positive sheep and goat sera (n = 46) from experimentally infected animals from Pirbright (UK), LCV (Mali) [23] and AHI (Ethiopia) [37], which were confirmed positive by a virus neutralization test (Supplementary Table S2); and (d) Capripox-negative sheep and goat sera (n = 216) from Austria (provided by AGES, Austria), where the disease is historically not present.

#### 2.4.2. Specificity Control Sera

Parapoxvirus-positive sera were used for a specificity study to exclude cross-reactivity. This consisted of thirteen samples from goats naturally infected with orf (Ecole Vétérinaire de Toulouse, Toulouse, France), six pseudocowpox (PCP)-positive cattle sera from Zambia, kindly provided by Maureen Ziba (CVRI, Zambia), and one bovine papular stomatitis (BPS) (kindly provided by AGES, Austria).

#### 2.4.3. Longitudinal Sera

We used two sets of samples from longitudinal studies. The first set comprised samples collected at 0, 6, 12, 18, 20, 23, 26, and 30 days post-infection (DPI) from two cattle experimentally infected with a virulent South African LSDV Neethling strain [36]. The second set consisted of samples collected at 0, 7, 14, 21, 28, 35, 42, 49, and 56 DPI from four goats that were infected experimentally with GTPV Oman 84. This was part of a larger SPPV/GTPV study with experimentally infected Ethiopian goats [37].

### 2.5. Development and Optimization of the CaPV iELISAs

#### 2.5.1. Antigen Coating

Serial dilution (chessboard) of the selected, purified A34 protein (GenScript, Piscataway, NJ, USA), diluted in 0.1 M carbonate/bicarbonate buffer and incubated overnight at 4 °C on a 96-well microtiter plate (Thermo Fisher Scientific, Waltham, MA, USA), was tested against several dilutions of sheep, cattle, and goat sera, and secondary antibodies (Merck KgaA, Darmstadt, Germany), to determine the optimal antigen amount for the iELISAs. For the optimization of antigen coating, the following antigen amounts per well were evaluated: 50 ng, 40 ng, 25 ng, 20 ng, 12.5 ng, and 10 ng. Two incubation times were tested: two hours and overnight.

#### 2.5.2. Effect of Blocking Buffers

Seven blocking buffers were compared to determine the optimal blocking conditions for the iELISAs. These buffers were: 5% milk in PBS 0.05% Tween 20, 5% BSA in PBS 0.05% Tween 20, Pierce Protein-Free T20 (BB1) (Thermo Fisher Scientific, Waltham, MA, USA), Pierce Protein-Free (PBS) (BB2) (Thermo Fisher Scientific, Waltham, MA, USA), Superblock T20 (BB3) (Thermo Fisher Scientific, Waltham, MA, USA), 10X Blocking Buffer (BB4) (Merck KgaA, Darmstadt, Germany), and ELISA Blocker (BB5) (Thermo Fisher Scientific, Waltham, MA, USA). The blocking buffers were used to block the plates and dilute the samples and secondary antibodies, as per the ELISA protocol details.

#### 2.5.3. Effect of Conjugates

Similarly, five conjugates were evaluated for the development of the iELISAs: anti-sheep-HRP (Merck KgaA, Darmstadt, Germany), anti-bovine IgG-peroxidase (Merck KgaA, Darmstadt, Germany), IDVet anti-ruminant conjugate (provided by IDVet, Montpellier, France), protein G–HRP (Merck KgaA, Darmstadt, Germany), and anti-goat/sheep-HRP GT-34 (SGB) (Merck KgaA, Darmstadt, Germany).

#### 2.5.4. LSD iELISA and SPP/GTP iELISA

Although the same antigen amount (25 ng/well) was used to coat the plates, we used different conditions depending on the animal origin of the serum. For bovine, we used the conditions we referred to as LSD iELISA; for sheep and goat sera, we used the conditions we referred to as SPP/GTP iELISA.

Coated plates were incubated for 30 min at room temperature with 50 µL/well of blocking buffer (Thermo Fisher Scientific, Waltham, MA, USA). After three washes of 250 µL/well each with PBS plus 0.05% Tween 20 (PBS-T), sera diluted in blocking buffer were added to the wells. For the LSD iELISA, 100 µL/well of cattle serum samples and controls were diluted 1 in 100. For the SPP/GTP iELISA, 100 µL/well of sheep or goat serum samples and controls were diluted 1 in 500. The plates were incubated with the sera at 37 °C for 90 min, and then washed as described above. Subsequently, the plates were incubated for 45 min at room temperature with 100 µL/well of secondary antibodies (Merck KgaA, Darmstadt, Germany), diluted 1 in 10,000 (the LSD iELISA) or 1 in 20,000 (the SPP/GTP iELISA) in a blocking buffer. The plates were washed with PBS-T as described above, and 100 µL/well of TMB substrate (Merck KgaA, Darmstadt, Germany) was added. After incubating the plates in the dark for 15 min, 100 µL/well of 1 M phosphoric acid (Merck KgaA, Darmstadt, Germany) was added to stop the reaction. The plates were read at 450 nm with a microplate reader, Multiskan Go (Thermo Fisher Scientific, Waltham, MA, USA).

### 2.6. Evaluation of Vaccinated Cattle Samples

As the assay performed on LSDV-vaccinated samples showed only borderline to weak positivity when using the above-described dilutions, we analyzed a panel of vaccinated serum (provided by the Scientific Veterinary Institute “Novi Sad”, Serbia) with an alternative protocol for vaccinated animals. The vaccinated sera samples were collected from cattle three months after vaccination with the Neethling vaccine (OBP) (n = 20). The serum status was established based on ELISA and/or VNT (range: 1 in 12 to 1 in 64). The samples were diluted 1 in 100, 1 in 10, and 1 in 2, and processed as described for other cattle sera. Forty-five negative cattle serum samples were also tested under the same ELISA conditions. For this sample panel, the average plus 3 and average plus 5 standard deviations were determined to establish thresholds.

### 2.7. Statistical Analysis

The ELISA raw OD values, relevant information about samples, and the VNT results were compiled in Microsoft Excel (Microsoft Corporation, Redmond, DC, USA). The background-subtracted OD values and the S/P% values ([Raw OD of sample/Raw OD Pos] ∗ 100) were calculated in Microsoft Excel, and these data were imported into R for further analysis. In addition to R base functions, the dplyr package and tidyr package were used for data frame manipulation and statistical analysis, respectively. The pROC package [38] was used for the ROC analysis, and the ggplot2 package [39] was used for the graphical representation of the data. Three methods: the Youden index = sensitivity + specificity-1 [40], the Euclidean index = (1 − sensitivity)^2^ + (1 − specificity)^2^ [41], and the product index = sensitivity * specificity [42], were used to determine the cut-off from the ROC analysis. The maximum values for the Youden index and the product index, and the minimum value for the Euclidean index, were used as a criterion for selecting the optimal cut-off point.

### 2.8. Institutional Review Board Statement

No animal experiments were carried out in the framework of this study. Serum samples were previously described or collected and submitted to the laboratories as part of routine diagnostic service and official surveillance programs, where according to national and EU legislations, ethical approval was not required.

## 3. Results

### 3.1. Expression and Analysis of the Recombinant Truncated C-Type Lectin-Like Glycoprotein A34 and the EEV Glycoprotein A36

Supplementary Figure S1a–c represents the images of a Western blot analysis of two recombinant truncated capripoxvirus proteins, A34 and A36. Both A34 and A36 reacted with the anti-penta His antibody, showing a single band at 18 kDa for A34 and a distinct band at around 28 kDa for A36 (Supplementary Figure S1a). In addition, both proteins reacted with LSD-positive serum, as shown in Supplementary Figure S1b. We also performed a Western blot probing with orf-positive serum to confirm the specificity. The result showed that A34 did not react with orf-positive serum. In contrast, a background reaction to orf serum was observed with A36 (Supplementary Figure S1c).

We therefore selected the truncated A34 protein for the development and optimization of the capripox iELISAs.

### 3.2. Optimization of the iELISAs

**Figure 1:**
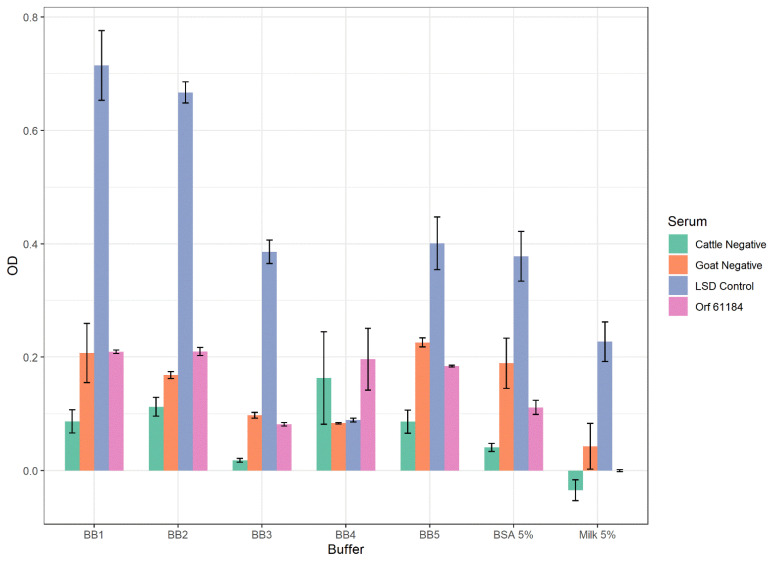
Comparative analysis of blocking buffers for the capripox iELISAs. Protein-based (5% milk and 5% BSA) and protein-free (BB1, BB2, BB3, BB4, and BB5) blocking buffer formulations were tested for the capripox iELISAs blocking, sera, and secondary antibody dilutions of LSD-positive and LSD-negative sera. While milk and BSA had low signals, BB1 produced low background and the highest positive signal. Data are presented as average of two runs in duplicate ± standard deviation.

In order to determine the optimal antigen amount per well to coat for the iELISAs, a serial dilution (chessboard) of purified A34 protein was tested against known dilutions of sheep, cattle, and goat sera, and secondary antibodies. The optimal antigen amount was estimated at 25 ng/well (Supplementary Figure S2 and Supplementary Table S3). To establish the best conditions for the iELISAs for CaPV antibody detection, we compared seven different types of blocking buffers: two protein-based buffers (5% milk in PBS-T and 5% BSA in PBS-T) and five protein-free buffers (BB1, BB2, BB3, BB4, and BB5). The result showed that BB1 (Figure 1 and Supplementary Table S4) produced the highest signal while maintaining an adequate signal-to-noise ratio. Therefore, this blocking buffer was selected for the optimization and analytical validation of the ELISAs. In addition, plate blocking, as well as sera and secondary antibody dilutions, were carried out using BB1. Next, we evaluated various secondary antibodies to select the most suitable one for the iELISAs. The monoclonal anti-goat/sheep IgG-peroxidase clone GT-34 (here referred to as SGB) provided the best signal-to-noise ratio when using sera from LSDV- or GTPV- and SPPV-positive animals. Based on the selected blocking buffer and secondary antibody, we determined the optimal dilution of sera and secondary antibody to detect anti-CaPV antibodies in cattle, sheep, and goats. The optimal serum dilution was 1:100 for cattle and 1:500 for sheep and goats (Supplementary Tables S5–S7). For the secondary antibody, the optimal dilution was 1:10,000 when using cattle sera, and 1:20,000 when using sheep or goat sera. We therefore needed different testing conditions for cattle serum and for sheep and goat sera when detecting antibodies against LSDV and SPPV/GTPV, respectively. Thus, we established one assay for anti-LSDV antibody detection in cattle (the LSD iELISA) and one for anti-SPPV and -GTPV antibody detection in sheep and goats (the SPP/GTP iELISA) with the above-described dilutions.

### 3.3. Cut-Off, Specificities, and Sensitivities of LSD iELISA and SPP/GTP iELISA

**Figure 2:**
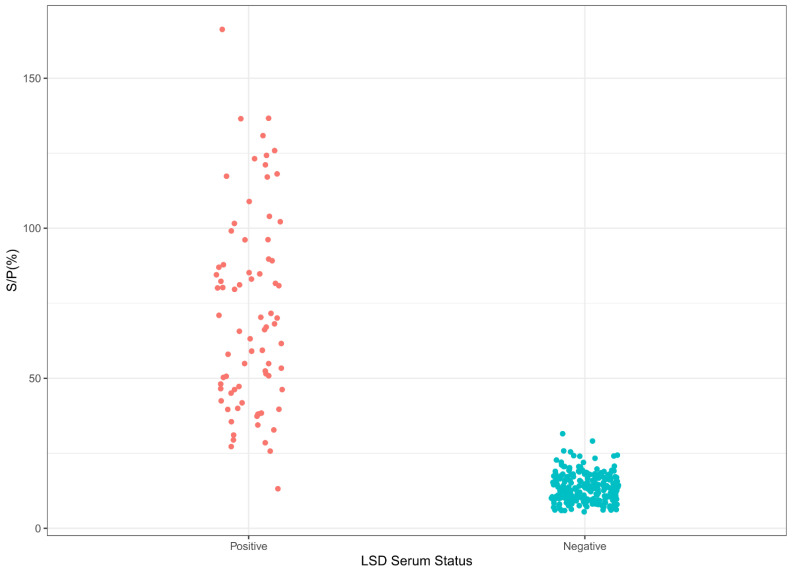
Sample distribution of the LSD iELISA. LSD-positive (78) and LSD-negative (252) serum samples were tested, using conditions for LSD iELISA.

The S/P% cut-off, diagnostic specificity (DSp), and diagnostic sensitivity (DSe) for the LSDV iELISA were obtained by analyzing the data resulting from testing our reference sample populations, consisting of 252 LSD-negative cattle and 78 LSD-positive cattle samples. Figure 2 presents the distribution of the S/P% values, showing good discrimination between the positive and negative serum populations. Based on the S/P% values, the cut-off for the LSD iELISA was 25.6%, using all three statistical approaches (Supplementary Figure S3). The ROC analysis estimated the area under the curve (AUC) to be 99.37% (Supplementary Figure S4), the calculated LSD iELISA sensitivity was 98.81%, and the specificity was 98.72%.

Likewise, the S/P% cut-off, diagnostic specificity (DSp), and diagnostic sensitivity (DSe) of the SPP/GTP iELISA were calculated using data from our reference populations, consisting of 216 negative sheep or goat serum and 46 positive sheep or goat serum samples.

**Figure 3:**
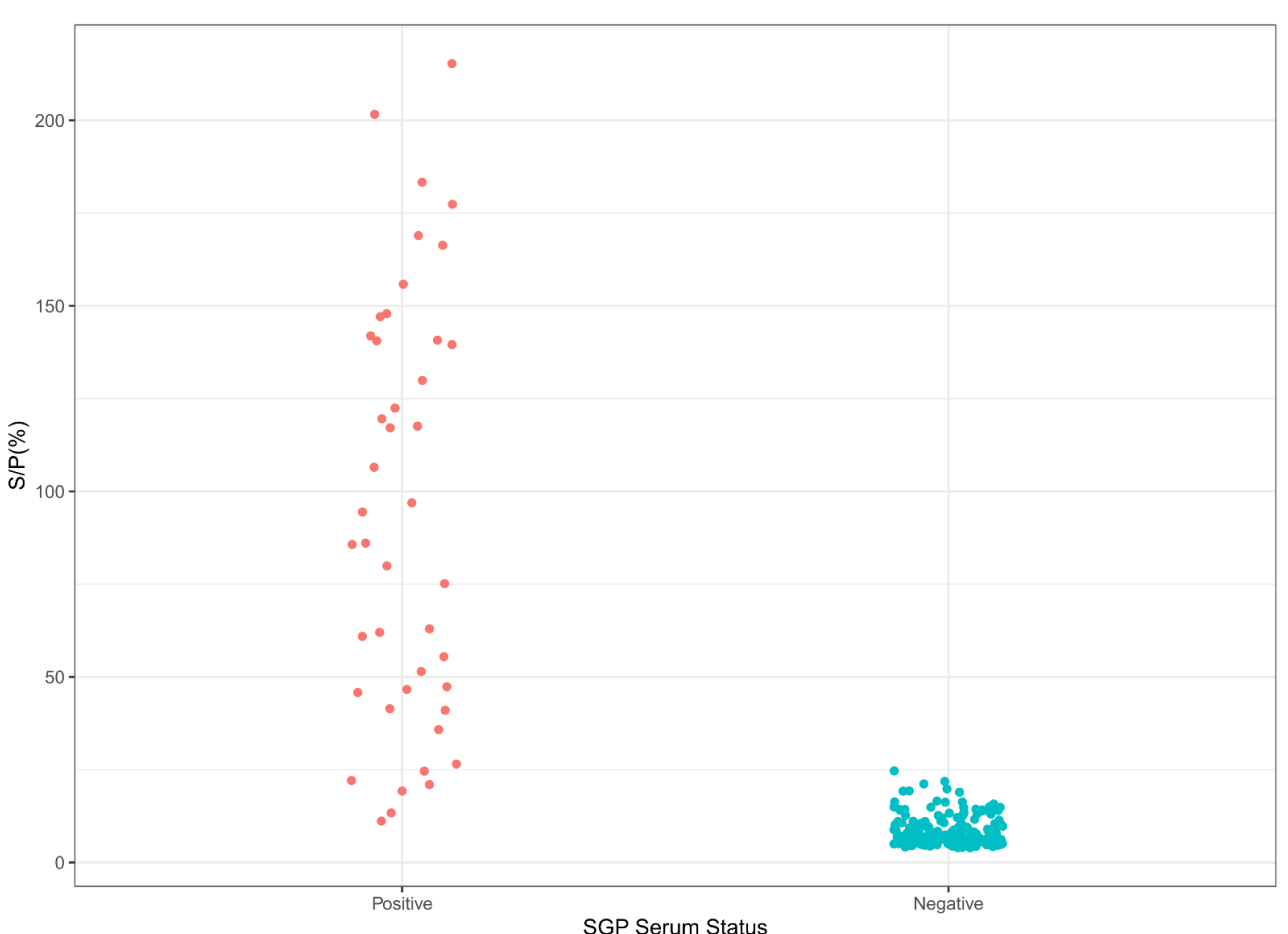
Sample distribution of the SGP iELISA. SPP- or GTP-positive (46) and -negative (216) serum samples were tested, using conditions for SPP/GTP iELISA.

These positive and negative populations were well-discriminated (Figure 3), though this discrimination was not as well-defined as for LSD. When the negative data were split according to animal species (sheep or goat), the separation of the S/P% between the positive and the negative populations was well-defined for sheep, while a higher background could be observed for goats (Supplementary Figure S5).

Based on the S/P% values, the cut-off for the SPP/GTP iELISA was 19.22%, using all three statistical approaches (Supplementary Figure S6).

The ROC analysis estimated the area under the curve (AUC) to be 99.18% (Supplementary Figure S7). The calculated LSD iELISA sensitivity was 97.65%, and the specificity was 95.35%.

### 3.4. Analysis of the Cross-Reactivity to Anti-Parapoxvirus Antibodies

Using the above-described protocols, we tested for specificity 13 orf-positive goat serum samples, as well as 6 pseudocowpoxvirus-positive and 1 bovine papular stomatitis virus-positive sera samples from cattle. Supplementary Figure S8 shows that all orf-positive samples tested negative in the SPP/GTP iELISA and had OD values comparable to negative sheep and goat samples. Similarly, all pseudocowpoxvirus and bovine papular stomatitis virus-positive sera tested negative in the LSD iELISA (OD values were from 0.0789 to 0.2529).

### 3.5. Antibody Detection in Sera from Longitudinal Studies on Experimentally Infected Animals

**Figure 4:**
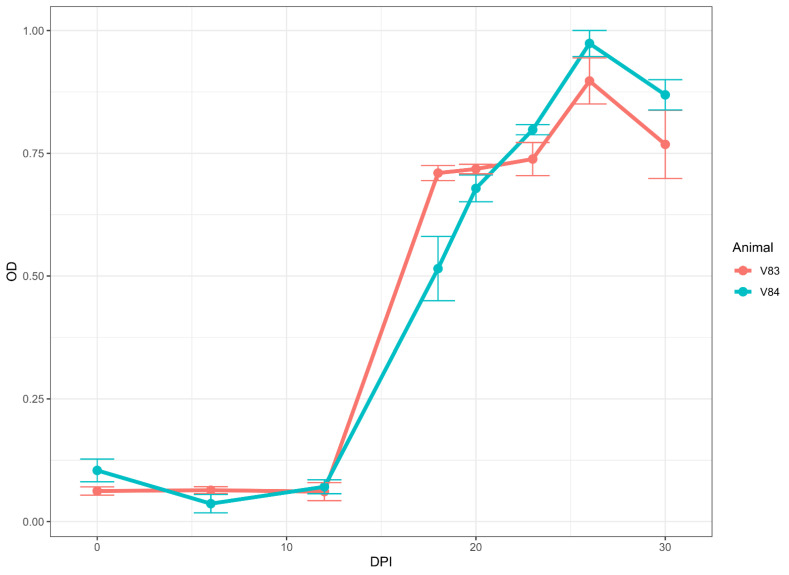
Immunoreactivity (seroconversion) of serum samples from cattle experimentally infected with virulent LSD isolate. (A) Two cattle were inoculated, and samples were collected at 0, 6, 12, 18, 20, 23, 26, and 30 days post-infection (DPI). Note the rise in immunoreactivity between 12 and 18 DPI for cattle. The data represent the average of two runs in duplicate ± standard deviation.

Once the conditions and cut-off values for the assay were established, we tested serum samples collected during two independent longitudinal studies with two different viruses. Figure 4 shows that for the two cattle infected with a virulent South African LSDV Neethling strain, the seroconversion occurred between 12 DPI and 18 DPI, corresponding to the expected two-week initial antibody response. Antibodies could be detected at every point after that, including the last collected data point (30 DPI).

**Figure 5:**
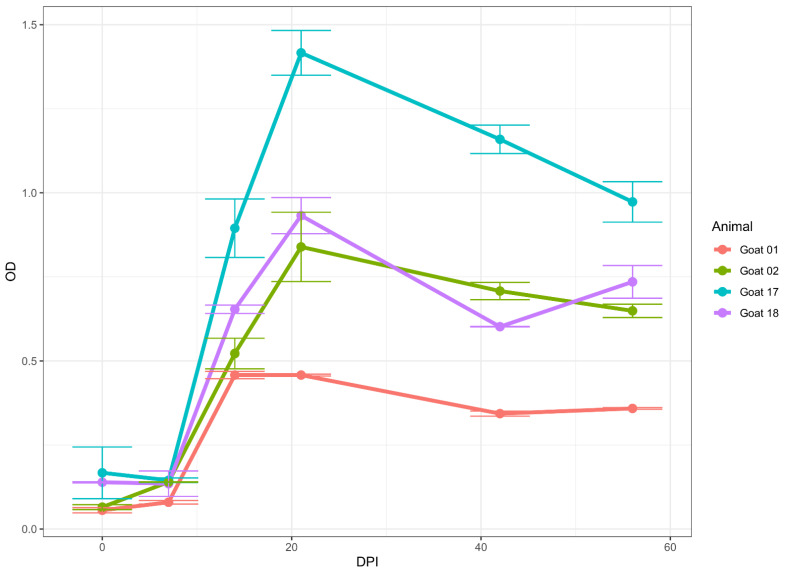
Immunoreactivity (seroconversion) of serum samples from goats experimentally infected with virulent GTPV Oman 84 isolates. Four goats were inoculated, and samples were collected at 7, 14, 21, 28, 35, 42, 49, and 56 DPI. Note that a raise in immunoreactivity was observed between 7 and 14 DPI for goats. The data represent the average of two runs in duplicate ± standard deviation.

Likewise, in four goats infected using GTPV Oman 84, seroconversion occurred between 7 DPI and 14 DPI (Figure 5). The goats remained positive from 14 DPI to the end of the collection at 56 DPI.

### 3.6. Performance of LSD iELISA on a Sample Panel of Serum from Vaccinated Cattle

When the vaccinated cattle samples were tested by the LSD iELISA, 20 out of 20 positive samples tested negative at 1/100 dilution. A total of 8 samples out of 20 (40%) tested positive for LSD in the iELISA at 1/10 dilution. Conversely, 90% of the samples (18 out of 20) were positive at a 1/2 dilution. The sample positivity was based on the cut-off determined from the 45 negative samples diluted 1 in 2. This suggests that the lower reactivity of vaccinated samples is probably due to low antibody production in vaccinated animals compared to the infected ones.

## 4. Discussion

In this study, we expressed and tested two recombinant truncated proteins for their use as antigens in an indirect ELISA to detect anti-capripoxvirus-specific antibodies. We selected two proteins, A34 and A36, based on previous work by Gelaye [34], who showed the immunoreactivity of these proteins with serum collected from capripoxvirus-positive animals. Following the expression, our study confirmed the reactivities of the two proteins with anti-capripoxvirus antibodies. However, due to cross-reactivity with orf-positive serum revealed by Western blot, we excluded A36 from further evaluation. Therefore, we used A34 as the only antigen to develop and optimize the indirect ELISA.

The A34 protein is a glycoprotein present in the outer membrane of the extracellular enveloped virion of poxviruses, involved in cell-to-cell transmission [43]. Previous studies on the antigenicity of vaccinia’s A34 protein showed its potential as a target candidate to develop an ELISA [44,45].

We first determined the optimum antigen amount for coating. Next, we compared various blocking buffers to optimize the test conditions and found that protein-free blocking buffers provided the highest signals and good signal-to-noise ratios, the best of them being BB1. Previous studies have also shown, based on the target antibodies, the usefulness of protein-free blocking buffers in both Western blots and ELISAs [46,47], as some of the high backgrounds observed in some ruminants’ sera may be due to their antibody reaction with ruminant proteins present in protein-based blocking buffers [47].

In our comparative analysis of various secondary antibodies, the SGB secondary antibody showed good reactivity to sheep, goat, and cattle serum. SGB is a monoclonal antibody raised in mice, against an epitope of the heavy chain of both goat IgG1 and IgG2.

The adequate reactivity of the SGB secondary antibody to cattle serum was surprising, as the manufacturer showed, on the accompanying data sheet for SGB, its strong affinity to sheep IgG and, to a much lesser degree, to bovine IgG. However, and as a consequence of this strong affinity, our further optimization resulted in a higher dilution of the SGB secondary antibody for both sheep and goat serum samples, as compared to cattle serum, to produce an equivalent response (signal-to-noise ratio). Similarly, the best dilution for sheep and goat sera was five times higher than for cattle serum.

Thus, based on these findings, we developed two different protocols depending upon the serum type used: one protocol for LSD (cattle serum) and one for SPP/GTP (sheep or goat serum).

The evaluation of these two protocols, using the relevant reference populations, enabled the establishment of a cut-off by using various statistical approaches and the determination of the diagnostic specificities and sensitivities. 

Although we calculated cut-off values using OD minus blank and S/N% (using both the positive and negative controls to compare runs), we settled for S/P% (using the positive control to compare runs). However, we found a strong agreement between OD values and S/P%.

As part of our longitudinal studies, we tested sera from an experimentally SPPV-infected Djelfa sheep, collected at 1, 7, 14, and 28 DPI, but due to the low volume of sera available, we could not generate statistical replicates for VNT and ELISA tests. The preliminary data for sheep showed antibody detection in sheep, starting at 14 DPI.

Our longitudinal analysis of serum collected from experimentally infected cattle, sheep, and goats, suggests that anti-capripoxvirus antibodies can be detected in all three species, starting at 14 DPI and to at least 56 DPI in goats. Unfortunately, for cattle and sheep, we could only detect antibodies up to 30 DPI and 28 DPI, respectively, since we did not have samples collected after those days.

Overall, the positive and negative sera data analysis showed better discrimination between LSD-positive and -negative samples than SPP- and GTP-positive and -negative samples. However, while focusing on the SPP/GTP-positive and -negative serum samples and analyzing them by species, positive and negative goat samples were less effectively discriminated as compared to positive and negative sheep samples. One explanation is that the SGB secondary antibody was raised against goats, thus producing a higher background due to a stronger affinity of the secondary antibody to the goat sera. This suggests a possible bias of the assay for one species versus the other; thus, one could consider different secondary antibody dilutions, or even develop alternative protocols for goats and sheep using different serum dilutions. A limitation of this study was the small number of sera from experimental trials tested; thus, further studies are needed to evaluate the antibody kinetics in infected animals. Another aspect that needs to be addressed is the detection of antibodies in vaccinated animals. We observed a weak detection of antibodies in vaccinated cattle under the current LSD iELISA protocol. We believe that this is a consequence of the low antibody titer produced by the vaccines, as only a localized infection occurs at the inoculation site [48]. Therefore, alternative protocols with increased sera or secondary concentrations would be required in such cases.

As with many indirect ELISAs, a weakness of our assays is that they are species-specific. Although our assays can detect anti-sheep, -goat, and -cattle antibodies, additional validation, for example, of wild ruminant sera, would be needed. Further changes in the ELISA format, such as developing a competitive ELISA based on the same target, would also help address the species-specificity issue.

The recent expansion of capripox diseases into new geographical regions has stressed the need for high-throughput tools for their surveillance [13,49].

Several ELISAs for capripoxvirus antibody detection have been reported. However, some use crude viral antigens, which renders them of high cost and not adequate to use in non-endemic countries [31]. For those based on recombinant proteins, mainly P32, several limitations exist, such as the inability to detect samples from natural outbreaks [32] or from vaccinated animals [33], or the difficulty in expressing, purifying, and preserving the antigen [50]. In addition, the evaluation included only a few samples from various origins [32].

A commercially available capripox ELISA kit (ID Screen^®^ Capripox Double Antigen Multi-species) has recently been introduced. The kit manufacturer claims a specificity greater than 99.2% on negative sheep, goat, and cattle sera. Although sensitivity validation data for LSD, ranging from 35.5–75% on vaccinated cattle, are readily available, there is limited information on the assay’s sensitivity for field-infected cattle and small ruminants [51,52,53]. According to an EFSA report, under experimental conditions, the sensitivity of the commercial ELISA evaluated within 1 month from infection or vaccination was 83%. In the same report, a sensitivity value of 59% was reported in the field for vaccinated animals [54]. Furthermore, the high concentrations of sera required for this assay make it less suitable for the surveillance of wildlife.

Contrasting with the shortcomings of many of the previously developed capripoxvirus ELISAs, our assays detected antibodies in the three species of domestic ruminants. Furthermore, their evaluation involved sera, confirmed positive by VNT, from various geographical origins. Additionally, the assays showed the potential to detect antibodies in vaccinated animals, although further studies will be needed. Finally, we confirmed the specificity of these assays regarding parapoxvirus sera.

In conclusion, our assays are highly sensitive and specific for detecting anti-capripoxvirus antibodies in sheep, goat, and cattle sera. Furthermore, they did not react with sera from naïve animals from disease-free countries or with sera positive for parapoxvirus.

Although further validation is required, these assays represent essential tools for trade-related screening, serosurveillance, epidemiological studies, and vaccination seromonitoring.

## Figures and Tables

**Table 1:** Primer sequences containing attB sites used in the amplification and cloning of the target regions for Gateway cloning.

Primer’s Name	Sequence (5′–3′)
C-type-GW-For	GGGGACAAGTTTGTACAAAAAAGCAGGCT**TA**ATACGATACAA AGATGAACTATTTCCTAATGTATGTAATAAAGGATGGG
C-type-GW-Rev	GGGGACCACTTTGTACAAGAAAGCTGGGT**A**TCAATTATATAA CTTTTAACACAGATTAT
EEVGp-GW-For	GGGGACAAGTTTGTACAAAAAAGCAGGCT**TA**GAATACAAAAAT GTTATTAAAAAAATGTTATTTAAA
EEVGp-GW-Rev	GGGGACCACTTTGTACAAGAAAGCTGGT**A**TTAACAACAA TTATAATAGTTTGACTCG

The underlined 25 nucleotides are attB sites followed by four “G” residues at the 5’ end; “TA” and “A” in bold are added, respectively, on the forward and reverse primers to maintain the frame shift; and the remaining residues next to the bold nucleotide/s are derived from gene sequence.

## Data Availability

The data presented in this study are available upon request from the corresponding author.

## Supplementary Materials

The following supporting information can be downloaded at: https://www.mdpi.com/article/10.3390/microorganisms10101956/s1, Figure S1: Western blot analysis of the truncated capripoxvirus; Figure S2: Optimization of purified A34 coated antigen; Figure S3: Estimation of cut**-**off values for LSD iELISA; Figure S4: Receiver operator curve analysis for LSD iELISA; Figure S5: Sample distribution of the SPP/GTP iELISA based on species; Figure S6: Estimation of cut-off values for SPP/GTP iELISA; Figure S7: Receiver operator curve analysis for SPP/GTP iELISA; Figure S8: Evaluation of analytical specificity of the SPP/GTP iELISA; Table S1: A34 ELISA and VNT results for LSD-positive samples; Table S2: A34 ELISA and VNT results for SPP- and GTP-positive samples; Table S3: A34 antigen per well against LSD-positive and -negative sera; Table S4: Blocking buffer optimization of LSD-positive and -negative sera; Table S5: Chessboard titration of SGB secondary antibody and serially diluted GTP-positive serum; Table S6: Chessboard titration of serially diluted SPP-positive serum; Table S7: Chessboard titration of SGB secondary antibody and serially diluted LSD-positive serum.
