# Supplementary material for: Development and Optimization of Indirect ELISAs for the Detection of Anti-Capripoxvirus Antibodies in Cattle, Sheep, and Goat Sera
Source: Microorganisms. 2022 Sep 30;10(10):1956. doi: 10.3390/microorganisms10101956 (PMC9608586; doi:10.3390/microorganisms10101956)
Supplement: Supplementary file 1 [file microorganisms-10-01956-s001.zip › microorganisms-1871040-supplementary.pdf]

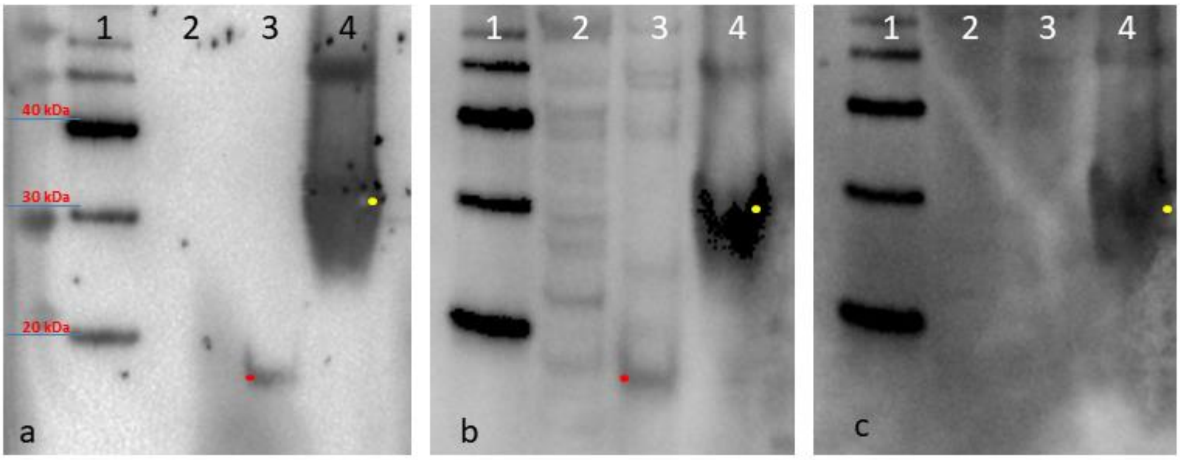

Supplementary Figure S1. Western blot analysis of the truncated capripoxvirus A34 and A36 proteins. The reactivity of the two proteins to anti-his antibodies, capripoxvirus specific and orf positive sera was evaluated. Lane 1. Molecular weight marker, lane 2. Cell lysate overexpressing NPPR (negative control), lane 3. Purified A34 (red dot), lane 4. Purified A36 (yellow dot). The membranes were probed with a. Anti-Penta-His antibody, b. Lumpy skin disease positive serum, c. Orf positive serum. While both proteins are detected by anti-Penta-His and LSD positive serum, only A36 reacted to orf positive serum. Note the presence of A34 band (line 3) and A36 band (line 4) in figure 1a and figure 1b and absence of the A34 band in figure 1c, indicating no crossreactivity of A34 with orf.

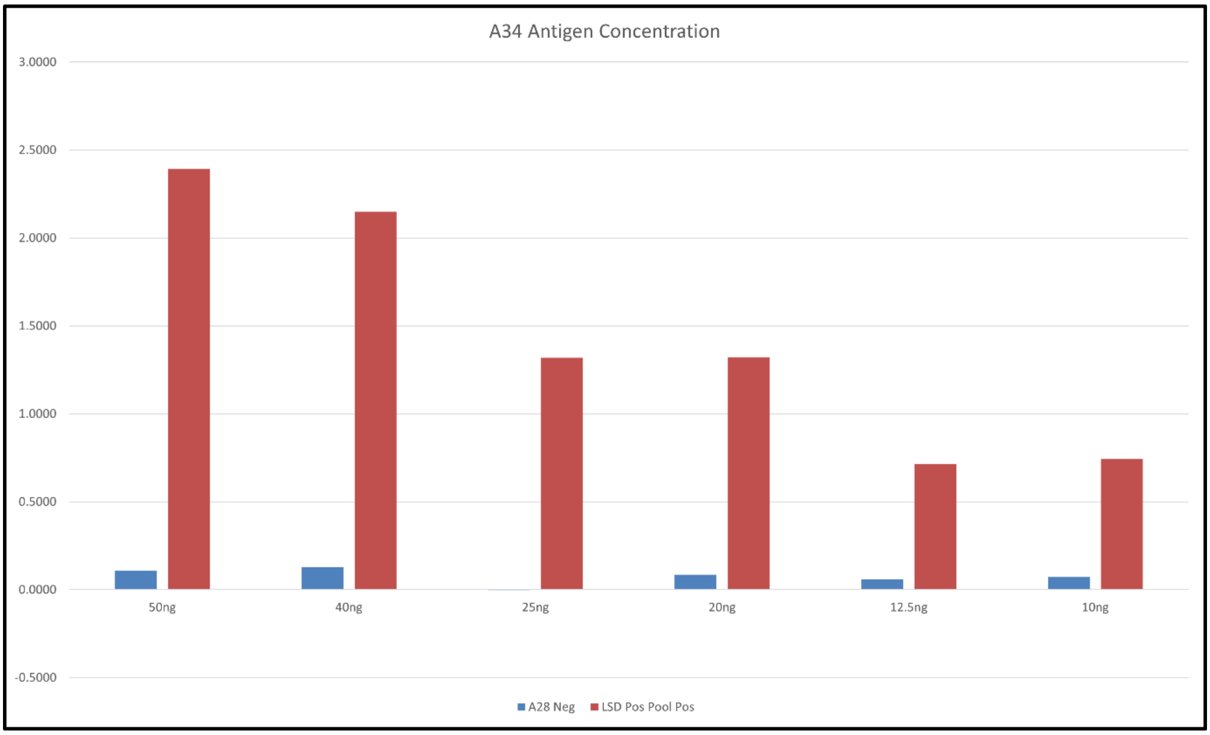

Supplementary Figure S2. Optimization of purified A34 coated antigen for the LSD iELISA. Maintaining the same sera and secondary antibody dilutions, the optimal antigen concentration for the A34 iELISA was estimated at 25ng/well.

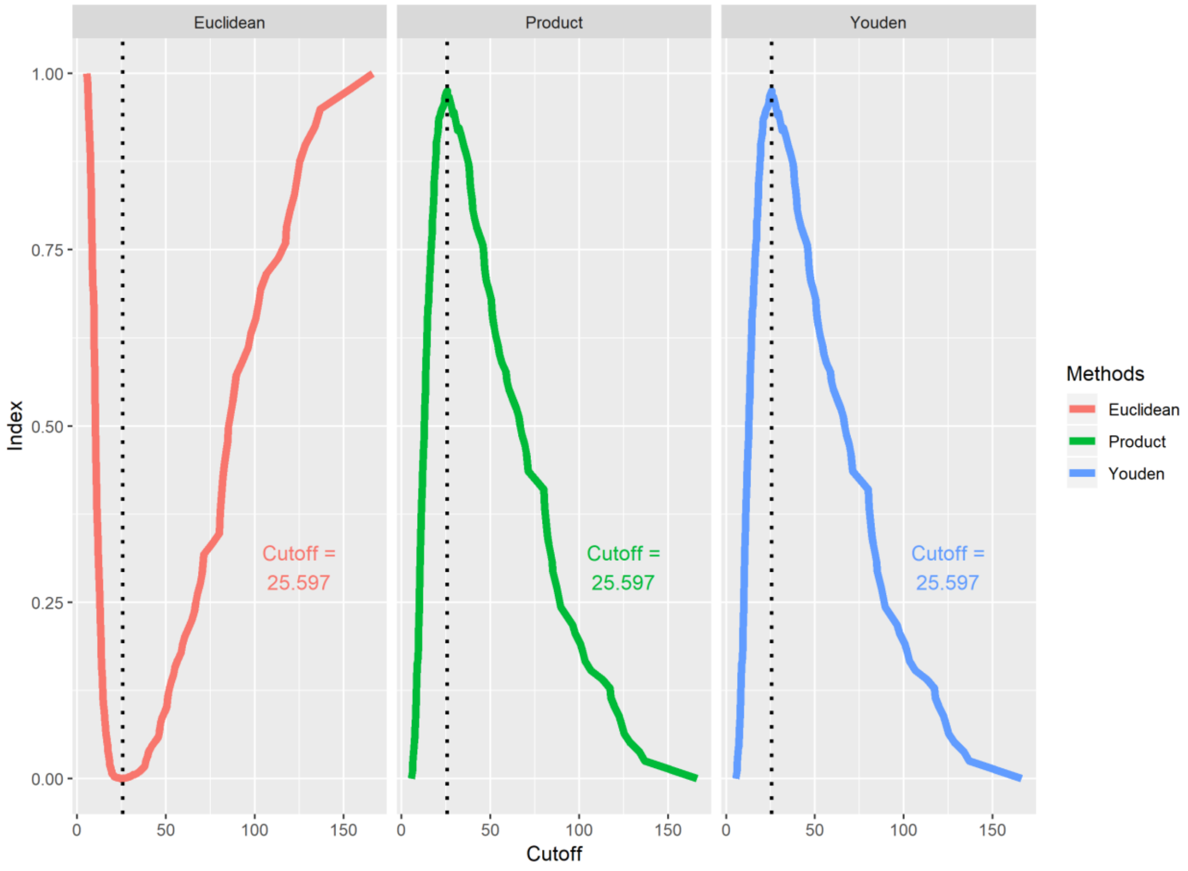

Supplementary Figure S3. Estimation of cut-off values for LSD iELISA. Three statistical indexes, Euclidean index Product index and Youden index were used to determine confidence intervals and cut-off value based on the calculated S/P%. All three indexes estimated a cut-off value, best diagnostic sensitivity and specificity of 25.6% for the LSD iELISA.

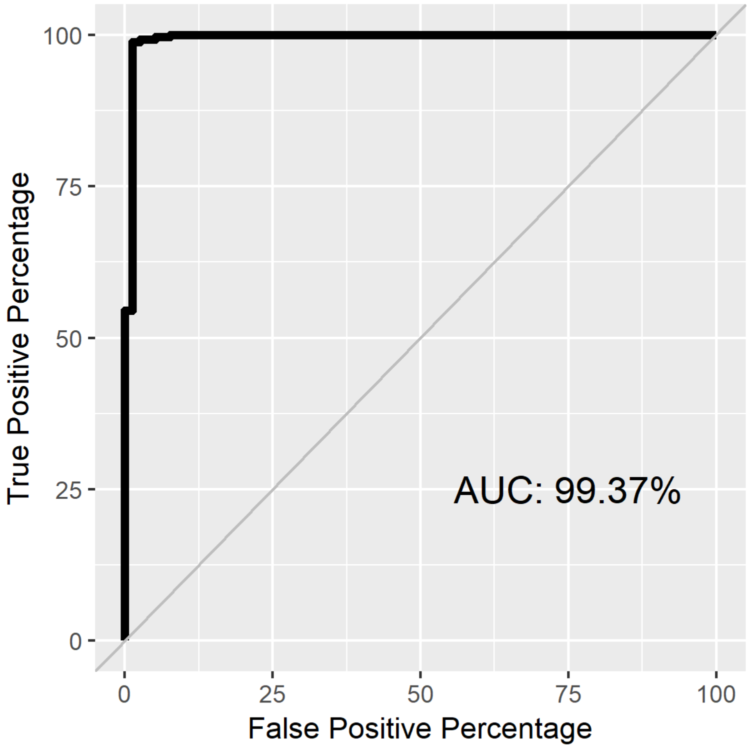

Supplementary Figure S4. Receiver operator curve analysis for LSD iELISA. The area under the curve for was 99.37%. Two diagnostic groups (VNT positive LSD samples/negative samples from LSD-free regions) were compared.

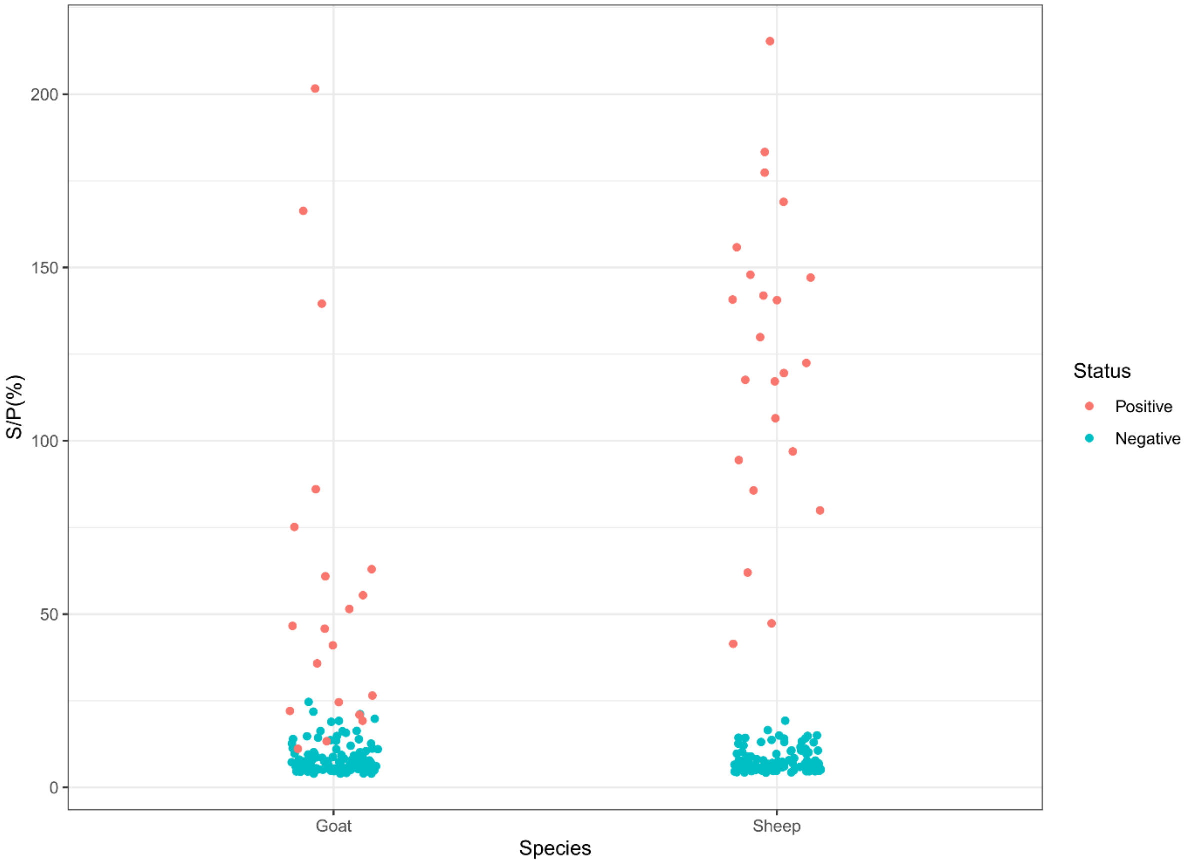

Supplementary Figure S5. Sample distribution of the SPP/GTP iELISA based on species. There was a better discrimination between sheep positive and negatives serum samples than goat positive and negative serum samples.

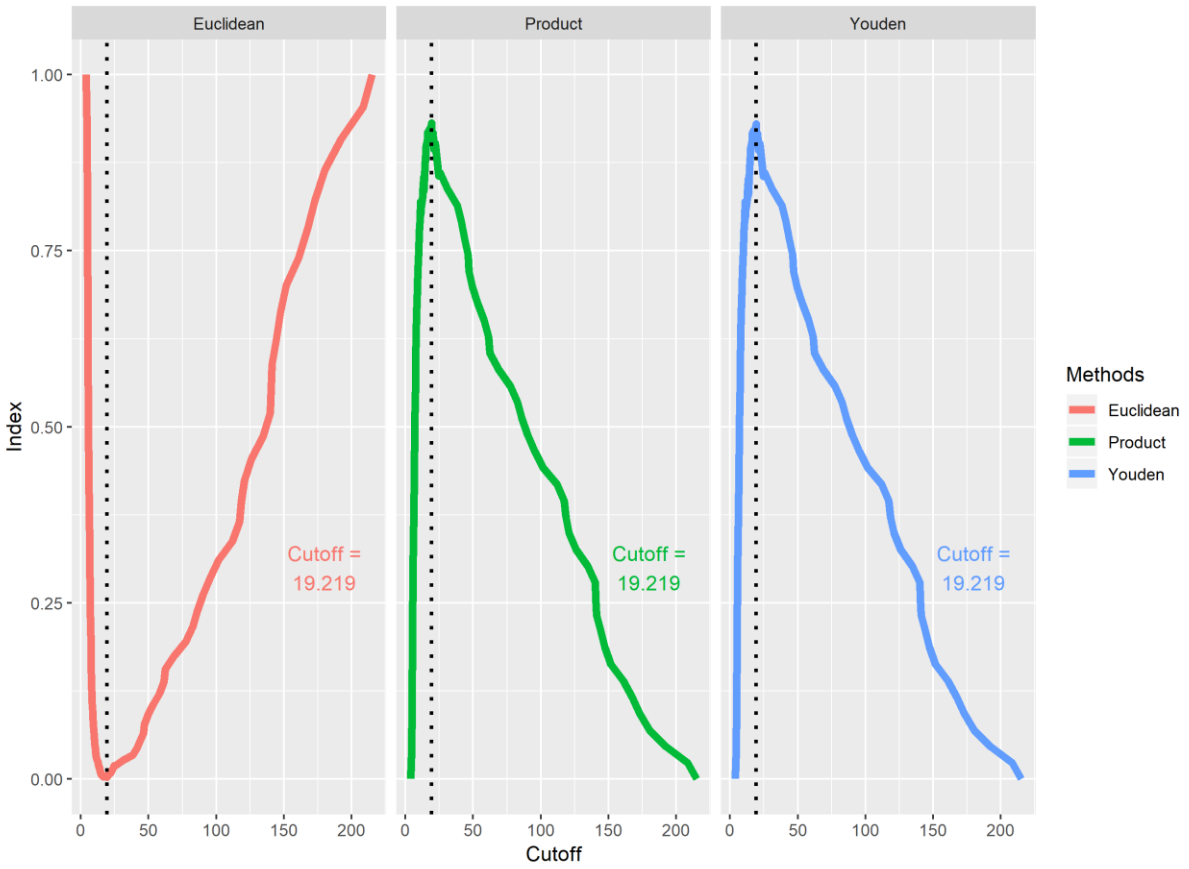

Supplementary Figure S6. Estimation of cut-off values for SPP/GTP iELISA. Three statistical indexes, Euclidean index, Product index and Youden index were used to determine confidence intervals and cut-off value based on the calculated S/P%. All three indexes estimated a cut-off value, best diagnostic sensitivity and specificity, of 19.2% for the SPP/GTP iELISA.

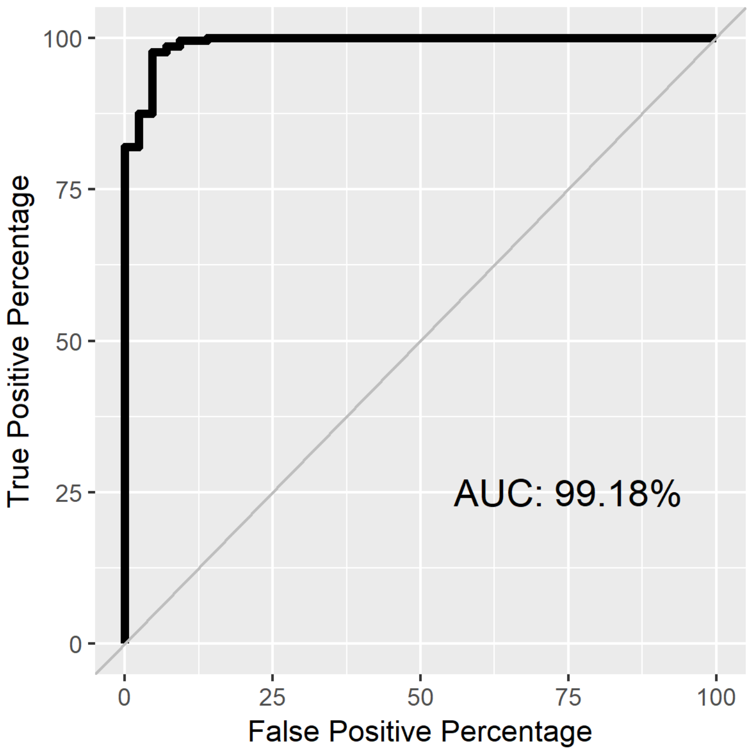

Supplementary Figure S7. Receiver operator curve analysis for SPP/GTP iELISA. The area under the curve was 99.18%. Two diagnostic groups (VNT positive LSD samples/negatives samples from SGP-free regions) were compared.

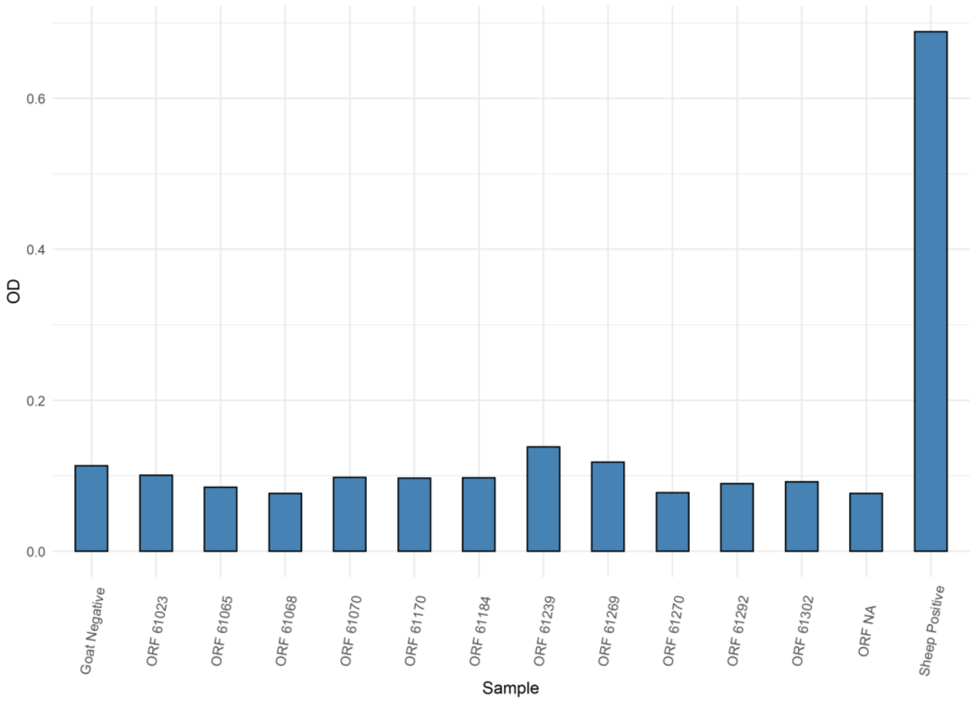

Supplementary Figure S8. Evaluation of analytical specificity of the SPP/GTP iELISA. Orf positive sera (12) were tested using the SPP/GTP iELISA. None of the orf positive samples tested positive (low optical density values were observed).

Supplementary Table S1. A34 ELISA and VNT results for LSD positive samples

SERUM_ID	A34 ELISA	A34 ELISA Results	VNT Dilution	VNT Result	S/P%-Neg	S/P% only Pos	OD minus BKG
**LSDV_MKD_382**	0.8382	Pos	1 in 16	Pos	57.725	68.11719899	0.8382
**LSDV_MKD_587**	0.7583	Pos	1 in 16	Pos	77.88534644	80.21404925	0.7583
**LSDV_MKD_666**	0.6917	Pos	1 in 16	Pos	56.70669454	63.13697893	0.6917
**Bul 031**	0.4708	Pos	1 in 16	Pos	35.25638088	42.48789026	0.4708
**Bul 055**	0.2836	Pos	1 in 16	Pos	18.10016955	27.24793422	0.2836
**Bul 068**	0.5816	Pos	1 in 16	Pos	45.41080511	51.50812065	0.5816
**Bul 096**	0.8124	Pos	1 in 16	Pos	66.56280071	70.29755363	0.8124
**Bul 098**	1.044	Pos	1 in 16	Pos	87.78811346	89.15211463	1.044
**Bul 113**	0.3719	Pos	1 in 16	Pos	26.19254915	34.43643913	0.3719
**Bul 140**	0.9902	Pos	1 in 16	Pos	82.85753563	84.77225546	0.9902
**Bul 141**	0.9953	Pos	1 in 16	Pos	83.32493241	85.18744657	0.9953
**Bul 236**	0.9598	Pos	1 in 16	Pos	80.07148421	82.29739081	0.9598
**Bul 328**	0.6776	Pos	1 in 16	Pos	54.20886221	59.32348272	0.6776
**Bul 329**	0.5933	Pos	1 in 16	Pos	46.48306832	52.4606179	0.5933
**Bul 330**	0.6237	Pos	1 in 16	Pos	49.26911974	54.93548256	0.6237
**Bul 048**	0.4250	Pos	1 in 16	Pos	24.61219832	37.35599888	0.425
**Bul 066**	1.5599	Pos	1 in 16	Pos	120.5503191	117.076426	1.5599
**Bul 195**	1.8366	Pos	1 in 64	Pos	143.940995	136.5130655	1.8366
**Bul 326**	1.0332	Pos	1 in 32	Pos	76.0260366	80.07867378	1.0332
**Bul 327**	0.5348	Pos	1 in 32	Pos	33.89407836	45.06883956	0.5348
**LSDV_MKD_013**	1.6624	Pos	1 in 250	Pos	129.2150978	124.2764822	1.6624
**LSDV_MKD_014**	0.5517	Pos	1 in 250	Pos	35.32271017	46.25597078	0.5517
**LSDV_MKD_016**	1.2625	Pos	1 in 250	Pos	95.40978063	96.18572633	1.2625
**LSDV_MKD_106**	1.7562	Pos	1 in 250	Pos	137.1444271	130.8654116	1.7562
**LSDV_MKD_107**	0.2594	Pos	1 in 8	Pos	10.61329727	25.72351784	0.2594
**LSDV_MKD_119**	1.3731	Pos	1 in 250	Pos	104.7592882	103.9547626	1.3731
**LSDV_MKD_161**	0.2991	Pos	1 in 250	Pos	13.969314	28.51222253	0.2991
**LSDV_MKD_164**	1.6468	Pos	1 in 250	Pos	127.8963608	123.1806687	1.6468
**LSDV_MKD_605**	0.7002	Pos	1 in 8	Pos	61.1737935	66.17100372	0.7002
**LSDV_MKD_662**	1.9122	Pos	1 in 250	Pos	176.0879871	166.2949195	1.9122
**LSDV_MKD_677**	1.0987	Pos	1 in 250	Pos	98.9570494	99.09128459	1.0987
**LSDV_MKD_678**	0.8869	Pos	1 in 32	Pos	78.87550962	81.59438249	0.8869
**LSDV_MKD_679**	1.3654	Pos	1 in 250	Pos	124.2438608	121.1235027	1.3654
**LSDV_MKD_170**	0.4168	Pos	1 in 16	Pos	31.39487226	38.4071303	0.4168
**LSDV_MKD_174**	0.5126	Pos	1 in 16	Pos	40.12022405	46.24064761	0.5126
**LSDV_MKD_176**	1.0108	Pos	1 in 16	Pos	85.49569653	86.97820843	1.0108
**LSDV_MKD_211**	0.4625	Pos	1 in 16	Pos	47.89903188	53.385334	0.4625
**LSDV_MKD_311**	0.4369	Pos	1 in 16	Pos	29.95129124	39.98897725	0.4369
**LSDV_MKD_313**	0.3804	Pos	1 in 16	Pos	24.75875379	35.54050862	0.3804
**LSDV_MKD_316**	0.4604	Pos	1 in 16	Pos	32.11101921	41.83922526	0.4604

**LSDV_MKD_357**	0.5725	Pos	1 in 16	Pos	42.41338112	50.66530194	0.5725
**LSDV_MKD_377**	0.7804	Pos	1 in 16	Pos	61.52008087	67.0340918	0.7804
**LSDV_MKD_383**	1.0995	Pos	1 in 16	Pos	83.855	87.82382443	1.0995
**LSDV_MKD_388**	0.7044	Pos	1 in 16	Pos	44.345	58.02632075	0.7044
**LSDV_MKD_391**	0.7511	Pos	1 in 16	Pos	49.015	61.54832384	0.7511
**LSDV_MKD_393**	0.8053	Pos	1 in 16	Pos	54.435	65.63595912	0.8053
**LSDV_MKD_394**	1.0069	Pos	1 in 16	Pos	74.595	80.84015234	1.0069
**LSDV_MKD_415**	0.4409	Pos	1 in 16	Pos	17.995	38.15377654	0.4409
**LSDV_MKD_429**	1.0551	Pos	1 in 16	Pos	79.415	84.47528187	1.0551
**LSDV_MKD_467**	0.6021	Pos	1 in 16	Pos	34.115	50.3110977	0.6021
**LSDV_MKD_534**	0.3482	Pos	1 in 16	Pos	25.14686461	32.79774316	0.3482
**LSDV_MKD_553**	0.3953	Pos	1 in 16	Pos	41.08672513	47.29037232	0.3953
**LSDV_MKD_563**	0.9208	Pos	1 in 16	Pos	25.14686461	32.79774316	0.3482
**LSDV_MKD_613**	0.4914	Pos	1 in 16	Pos	37.23078419	46.55379393	0.4914
**LSDV_MKD_621**	0.3047	Pos	1 in 16	Pos	19.07725218	31.09657656	0.3047
**LSDV_MKD_629**	0.5102	Pos	1 in 16	Pos	39.05877777	48.11027859	0.5102
**LSDV_MKD_631**	0.0883	Neg	1 in 16	Pos	-1.96412076	13.18044459	0.0883
**LSDV_MKD_639**	1.0901	Pos	1 in 16	Pos	95.4446011	96.1212071	1.0901
**LSDV_MKD_672**	0.4121	Pos	1 in 16	Pos	30.96680177	38.02281369	0.4121
**LSDV_MKD_674**	0.4325	Pos	1 in 16	Pos	32.82480987	39.69091132	0.4325
**LSDV_MKD_689**	0.6422	Pos	1 in 16	Pos	51.89362633	59.03878793	0.6422
**LSDV_MKD_699**	0.6187	Pos	1 in 16	Pos	49.78368778	54.91639069	0.6187
**LSDV_MKD_042**	1.6852	Pos	1 in 250	Pos	131.1424828	125.8780556	1.6852
**LSDV_MKD_062**	0.8904	Pos	1 in 250	Pos	63.95452048	70.04776623	0.8904
**LSDV_MKD_118**	1.3476	Pos	1 in 250	Pos	102.6036603	102.1635291	1.3476
**LSDV_MKD_120**	0.9033	Pos	1 in 8	Pos	65.04501458	70.95391964	0.9033
**LSDV_MKD_180**	1.5746	Pos	1 in 250	Pos	121.7929752	118.1090194	1.5746
**LSDV_MKD_187**	0.6171	Pos	1 in 250	Pos	40.85126168	50.84995785	0.6171
**LSDV_MKD_251**	1.3393	Pos	1 in 250	Pos	101.9020246	101.5805001	1.3393
**LSDV_MKD_475**	1.0753	Pos	1 in 32	Pos	79.58493597	83.03596516	1.0753
**LSDV_MKD_488**	0.3125	Pos	1 in 8	Pos	15.10207532	29.45349817	0.3125
**LSDV_MKD_489**	0.4574	Pos	1 in 16	Pos	27.35111374	39.63191908	0.4574
**LSDV_MKD_493**	1.3196	Pos	1 in 64	Pos	119.9013938	117.3399422	1.3196
**LSDV_MKD_535**	0.881	Pos	1 in 250	Pos	78.31610885	81.10698059	0.881
**LSDV_MKD_650**	0.766	Pos	1 in 64	Pos	67.41253437	71.60677406	0.766
**LSDV_MKD_658**	1.5535	Pos	1 in 250	Pos	142.0783161	136.6625361	1.5535
**LSDV_MKD_665**	1.2176	Pos	1 in 250	Pos	110.2303973	108.913672	1.2176
**LSDV_MKD_692**	0.9851	Pos	1 in 32	Pos	88.18621409	89.70673276	0.9851

Supplementary Table S2. A34 ELISA and VNT results for SPP and GTP positive samples

ORIGIN	Species	Sample ID	A34 OD minus BKG	Result	VNT Dilution	VNT Results	S/P%-Neg	S/P%only
Djelfa	Goat	Djelfa Goat 10 D28	0.216	Pos	1 in 16	Pos	17.63536	24.59739
Djelfa	Goat	Djelfa Goat 11 D21	0.35415	Pos	1 in 16	Pos	29.84751	35.77729
Djelfa	Sheep	Djelfa sheep 01 D28	0.5215	Pos	1 in 16	Pos	45.99777	47.33594
Djelfa	Sheep	Djelfa sheep 02 D14	0.4564	Pos	1 in 16	Pos	39.93857	41.42689
Djelfa	Sheep	Djelfa sheep 06 D28	0.6781	Pos	1 in 16	Pos	58.48398	61.9932
NAHDIC	Goat	DPI 14 Goat 15 Group 1 S10	0.14975	Pos	1 in 16	Pos	11.77901	19.23606
NAHDIC	Goat	DPI 28 Goat 2944 Group 1 S08	0.2560	Pos	1 in 16	Pos	3.754878	45.79066
NAHDIC	Goat	DPI 28 Goat 19 Group 1 S08	0.1846	Pos	1 in 16	Pos	14.85967	22.05632
NAHDIC	Goat	DPI 14 Goat 28 Group 1 S10	0.2395	Pos	1 in 16	Pos	19.71271	26.49915
NAHDIC	Goat	DPI 21 Goat 21 Group 1 S06	0.231	Pos	1 in 16	Pos	18.95942	20.9676
NAHDIC	Goat	DPI28 Goat 17 Group 5 S08	1.3287	Pos	1 in 16	Pos	111.0249	201.6271
**NAHDIC**	**Goat**	**DPI 14 Goat 24 Group 2 S10**	**0.0915**	Neg	1 in 16	Pos	**5.330387**	**13.33252**
NAHDIC	Goat	DPI 28 Goat 10 Group 2 S08	0.2230	Pos	1 in 16	Pos	0.454878	40.99659
NAHDIC	Goat	DPI 28 Goat 05 Group 2 S08	0.2951	Pos	1 in 16	Pos	7.664878	51.47091
**NAHDIC**	**Goat**	**DPI 28 Goat 2949 Group 2 S08**	**0.1418**	Neg	1 in 16	Pos	**2.943646**	**11.14753**
NAHDIC	Goat	DPI 28 Goat 01 Group 5 S08	0.5331	Pos	1 in 16	Pos	31.46488	86.04634
NAHDIC	Goat	DPI 28 Goat 12 Group 3 S08	0.3601	Pos	1 in 16	Pos	14.16488	60.91378
NAHDIC	Goat	DPI 28 Goat 27 Group 3 S08	0.2616	Pos	1 in 16	Pos	4.314878	46.6042
NAHDIC	Goat	DPI 28 Goat 2942 Group 3 S08	0.3225	Pos	1 in 16	Pos	10.40488	55.45144
NAHDIC	Goat	DPI 21 Goat 2937 Group 3 S06	0.8279	Pos	1 in 16	Pos	74.51601	75.1475

NAHDIC	Goat	DPI 28 Goat 9 Group 3 S08	0.6899	Pos	1 in 16	Pos	59.52707	62.94813
NAHDIC	Goat	DPI 28 Goat 2 Group 5 S08	0.9015	Pos	1 in 16	Pos	68.30488	139.5656
NAHDIC	Goat	DPI 28 Goat 18 Group 5 S08	1.0857	Pos	1 in 16	Pos	86.72488	166.3253
Pirbright	Sheep	Mong Pirbright P1/07-01	0.4909	Pos	Test VNT positive at Pirbright	Pos	27.24488	79.91574
Pirbright	Sheep	Mong Pirbright P1/07-02	0.9176	Pos	Test VNT positive at Pirbright	Pos	69.91488	141.9046
Pirbright	Sheep	Mong Pirbright P1/07-03	0.6739	Pos	Test VNT positive at Pirbright	Pos	45.54488	106.5011
Pirbright	Sheep	Mong Pirbright P1/07-04	0.5909	Pos	Test VNT positive at Pirbright	Pos	37.24488	94.44323
Pirbright	Sheep	Mong Pirbright P1/07-05	1.2027	Pos	Test VNT positive at Pirbright	Pos	98.42488	183.3224
Pirbright	Sheep	Mong Pirbright P1/07-06	0.7837	Pos	Test VNT positive at Pirbright	Pos	56.52488	122.4522
Pirbright	Sheep	Mong Pirbright P1/07-07	1.1617	Pos	Test VNT positive at Pirbright	Pos	94.32488	177.3662
Pirbright	Sheep	Mong Pirbright P1/07-08	0.7471	Pos	Test VNT positive at Pirbright	Pos	52.86488	117.1352
Pirbright	Sheep	Mong Pirbright P1/07-09	1.0135	Pos	Test VNT positive at Pirbright	Pos	79.50488	155.8364
Pirbright	Sheep	Mong Pirbright P1/07-10	1.4228	Pos	Test VNT positive at Pirbright	Pos	120.4349	215.2975
Pirbright	Sheep	Mong Pirbright P2/08-01	0.9097	Pos	Test VNT positive at Pirbright	Pos	69.12488	140.7569
Pirbright	Sheep	Mong Pirbright P2/08-02	0.9085	Pos	Test VNT positive at Pirbright	Pos	69.00488	140.5826
Pirbright	Sheep	Mong Pirbright P2/08-03	1.1037	Pos	Test VNT positive at Pirbright	Pos	88.52488	168.9402
Pirbright	Sheep	Mong Pirbright P2/08-04	0.8350	Pos	Test VNT positive at Pirbright	Pos	61.65488	129.9048
Pirbright	Sheep	Mong Pirbright P2/08-05	0.9533	Pos	Test VNT positive at Pirbright	Pos	73.48488	147.0909

Pirbright	Sheep	Mong Pirbright P2/08-06	0.7637	Pos	Test VNT positive at Pirbright	Pos	54.52488	119.5467
Pirbright	Sheep	Mong Pirbright P2/08-07	0.5306	Pos	Test VNT positive at Pirbright	Pos	31.21488	85.68316
Pirbright	Sheep	Mong Pirbright P2/08-08	0.7502	Pos	Test VNT positive at Pirbright	Pos	53.17488	117.5855
Pirbright	Sheep	Mong Pirbright P2/08-09	0.9589	Pos	Test VNT positive at Pirbright	Pos	74.04488	147.9044
Pirbright	Sheep	Mong Pirbright P2/08-10	0.6080	Pos	Test VNT positive at Pirbright	Pos	38.95488	96.92744
Pirbright	Sheep	RW 35 21 DPI	0.2812	Pos	Test VNT positive at Pirbright	Pos	6.274878	49.45159
Pirbright	Sheep	SPPV Morocc Vac 291 D28	0.3169	Pos	Test VNT positive at Pirbright	Pos	9.844878	54.6379
Pirbright	Sheep	SPPV Morocc Vac 8328	0.2898	Pos	Test VNT positive at Pirbright	Pos	7.134878	50.70095

Supplementary Table S3. A34 antigen per well against LSD positive and negative sera

		A34 Antigen Amount					
Sample Code	Sample Type	50ng	40ng	25ng	20ng	12.5ng	10ng
BH32	Neg	0.1330	0.28585	0.0392	0.0922	0.0637	0.0567
BH36	Neg	0.2565	0.36735	0.0677	0.1378	0.1050	0.0857
BH37	Neg	0.2220	0.42115	0.16665	0.1847	0.0950	0.1351
BH05	Neg	0.2896	0.20905	0.04205	0.1544	0.0929	0.0659
BH30	Neg	0.0755	0.1705	0.00085	0.0717	0.0205	0.0734
A28	Neg	0.1076	0.1283	-0.002	0.0863	0.0596	0.0735
LSD Pos Pool	Pos	2.3933	2.14985	1.31805	1.3231	0.7162	0.7436

Supplementary Table S4. Blocking buffer optimization of LSD positive and negative sera. Milk, BSA and five non-protein-based buffers were used.

Sample	Buffer	Avg	STD
LSD Control	BB1	0.71465	0.061589
Cattle Negative	BB1	0.0867	0.020365
Orf 61184	BB1	0.20935	0.003041
Goat Negative	BB1	0.2073	0.052184
LSD Control	BB2	0.66695	0.018738
Cattle Negative	BB2	0.11255	0.016758
Orf 61184	BB2	0.21005	0.007142
Goat Negative	BB2	0.16845	0.006293
LSD Control	BB3	0.3857	0.020648
Cattle Negative	BB3	0.01805	0.003182
Orf 61184	BB3	0.08175	0.003182
Goat Negative	BB3	0.0976	0.005233
LSD Control	BB4	0.0892	0.003394
Cattle Negative	BB4	0.1632	0.081317
Orf 61184	BB4	0.1963	0.054306
Goat Negative	BB4	0.0835	0.001273
LSD Control	BB5	0.40055	0.046457
Cattle Negative	BB5	0.0862	0.020365
Orf 61184	BB5	0.1844	0.001556
Goat Negative	BB5	0.22585	0.007849
LSD Control	BSA 5%	0.3779	0.043982
Cattle Negative	BSA 5%	0.04075	0.007142
Orf 61184	BSA 5%	0.11145	0.012657
Goat Negative	BSA 5%	0.18925	0.044053
LSD Control	Milk 5%	0.2272	0.034507
Cattle Negative	Milk 5%	-0.03475	0.018597
Orf 61184	Milk 5%	-0.0002	0.001838
Goat Negative	Milk 5%	0.0429	0.040447

Supplementary Table S5. Chessboard titration of SGB secondary antibody and serially diluted GTP positive serum.

Sample	Serum	Conjugate	OD	Ratio Pos/Neg
GTP_Pos	1 in 50	1 in 5k	2.4135	10.34949
GTP_Pos	1 in 100	1 in 5k	1.797	9.776931
GTP_Pos	1 in 200	1 in 5k	1.3035	13.06112
GTP_Pos	1 in 400	1 in 5k	1.0419	23.102
GTP_Pos	1 in 800	1 in 5k	0.7741	29.21132
GTP_Pos	1 in 50	1 in 10k	1.8974	7.989053
GTP_Pos	1 in 100	1 in 10k	1.5136	10.21323
GTP_Pos	1 in 200	1 in 10k	1.1903	10.40472
GTP_Pos	1 in 400	1 in 10k	0.9335	17.51407
GTP_Pos	1 in 800	1 in 10k	0.7475	47.31013
GTP_Pos	1 in 50	1 in 20k	1.8218	11.90719
GTP_Pos	1 in 100	1 in 20k	1.402	14.75789
GTP_Pos	1 in 200	1 in 20k	1.0189	12.57901
GTP_Pos	1 in 400	1 in 20k	0.8538	23.01348
GTP_Pos	1 in 800	1 in 20k	0.6502	92.88571
GTP_Pos	1 in 50	1 in 40k	1.4226	8.87461
GTP_Pos	1 in 100	1 in 40k	1.0587	19.17935
GTP_Pos	1 in 200	1 in 40k	0.8149	10.22459
GTP_Pos	1 in 400	1 in 40k	0.6965	25.05396
GTP_Pos	1 in 800	1 in 40k	0.5223	44.64103

Supplementary Table S6. Chessboard titration of serially diluted SPP positive serum. SGB secondary antibody fixed at 1 in 20k in BB1

	Sheep Pos	Neg
1 in 100	1.612133	0.113233
1 in 150	1.464533	0.077133
1 in 200	1.265733	0.064933
1 in 300	1.233333	0.040833
1 in 400	1.075333	0.045733
1 in 600	1.074633	0.027233
1 in 800	0.904433	0.022233

Supplementary Table S7. Chessboard titration of SGB secondary antibody and serially diluted LSD positive serum. Final LSD sera (1 in 100) and SGB (1in 10k) dilutions were selected after additional testing of high background negative sera.

	LSD Pos	1 in 25		1 in 50		1 in 100		1 in 200		1 in 400	
		Avg	Pos/Neg Ratio	Avg	Pos/Neg Ratio	Avg	Pos/Neg Ratio	Avg	Pos/Neg Ratio	Avg	Pos/Neg Ratio
SGB	1 in 5k	0.820	8.067	0.749	9.394	0.689	7.676	0.752	10.648	0.600	7.744
	1 in 10k	0.765	8.852	0.699	10.898	0.624	9.019	0.588	6.838	0.609	8.197
	1 in 20k	0.563	9.086	0.581	9.780	0.546	9.234	0.556	10.037	0.551	9.883
